# Public Discourse on Menopausal Skin Management: A YouTube Infodemiology Study of Treatment Perceptions, Sentiment, and Unmet Needs Across 43 954 Viewer Comments

**DOI:** 10.1111/jocd.71101

**Published:** 2026-07-27

**Authors:** Esin Diremsizoglu, Emre Akif Arslan, Abdullah Demirbas, Sabrina G. Fabi

**Affiliations:** ^1^ Kocaeli University Faculty of Medicine Kocaeli Turkiye; ^2^ TEV Inanc High School Istanbul Turkiye; ^3^ University of California San Diego Medical Center San Diego California USA

**Keywords:** cosmeceuticals, hormone replacement therapy, infodemiology, menopause, patient information, public discourse, sentiment analysis, skin aging, social media, youtube

## Abstract

**Background:**

Menopausal skin changes carry significant psychosocial burden yet remain clinically under‐addressed. As women increasingly turn to digital platforms for information and peer experience, YouTube viewer comments represent an underexplored source of real‐world treatment perceptions at scale.

**Methods:**

Cross‐sectional infodemiology study using the YouTube Data API v3 (14 March 2026). 342 videos yielded 43 954 analyzable comments after automated cleaning (350‐comment/video cap). Videos were classified into four content‐type categories (Cohen's κ = 0.934). A 13‐category management subcorpus (*n* = 10 861; 24.7% of full corpus) was constructed by pre‐specified keyword matching.

**Results:**

The study corpus showed predominantly positive sentiment (59.6% positive, 19.1% negative; median 0.36, IQR 0.00–0.73). Hormone replacement therapy (HRT) dominated the management subcorpus (54.8%) yet generated the lowest sentiment of any category (median 0.10; 30.2% negative), differing significantly from all others (*p* < 0.001). Alternative approaches (median 0.72; 77.7% positive) and supplements (median 0.50; 74.6% positive) attracted the highest positivity. Personal/lifestyle content generated higher positivity (median 0.59; 70.6%) than clinician content (median 0.32; 57.7%; *p* < 0.001). Following the FDA's November 2025 HRT boxed warning removal, a higher proportion of positive HRT comments was observed in the post‐announcement period (53.1% vs. 50.5%; *p* = 0.126) over the 4.5‐month available window. A negative temporal trend was observed over 116 months (Spearman ρ = −0.424, *p* < 0.001).

**Conclusions:**

HRT generates the most negative public commentary of any management category, while supplements and alternative approaches attract positivity disproportionate to their evidence base. Clinician content elicits lower viewer positivity than lifestyle creators. Dermatologists should address HRT safety misconceptions and increase online representation of evidence‐based procedural options.

## 1 Introduction

Menopausal estrogen deficiency results in cutaneous changes, including collagen loss, reduced dermal thickness, xerosis, and progressive facial laxity, that carry substantial psychosocial burden yet remain consistently underaddressed in clinical menopause care [1, 2]. Hormone therapy, topical retinoids, and energy‐based devices demonstrate measurable benefit for hormone‐related skin changes; however, no treatment carries a specific regulatory indication for estrogen‐deficient skin, and current clinical guidelines do not endorse hormone therapy on dermatological grounds alone [2, 3]. This evidence gap is compounded by a rapidly expanding commercial landscape of unregulated nutraceuticals and topical cosmeceuticals that position themselves as alternatives to evidence‐based care [4, 5].

As formal clinical guidance remains limited, women increasingly turn to YouTube, one of the most widely used online health information platforms globally, to seek information and share lived experience [6]. Infodemiology offers a validated framework for extracting real‐world patient insights from unsolicited user‐generated content [7, 8]. Comment‐centred infodemiology has identified misinformation patterns and unmet needs across comparable chronic and hormonal conditions and captures a patient perspective that structured clinical research substantially underrepresents [8, 9]. Within dermatology, however, social media research has focused almost exclusively on video quality metrics rather than viewer commentary, and no prior study has applied this approach to menopausal skin [10, 11]. The FDA's November 2025 removal of longstanding HRT boxed warnings provides timely context to examine whether regulatory shifts are reflected in public discourse [12].

Understanding how patients perceive and discuss treatment options in unmoderated digital spaces may inform both clinical communication strategies and the prioritization of evidence‐based content online. The present study therefore characterizes public discourse on menopausal skin management across YouTube, with a focus on treatment perceptions, sentiment distribution, and unmet informational needs.

## 2 Methods

### 2.1 Ethical Considerations

No ethics approval was required as all data were publicly available. Usernames were not recorded; any identifiable information was pseudonymised prior to analysis.

### 2.2 Data Extraction

All data were extracted on 14 March 2026 via the YouTube Data API v3. Videos were identified using a pre‐specified query bank combining terms across three domains: hormonal transition, skin quality, and treatment/management. Queries were English‐language only and deduplicated by video ID, yielding 663 unique videos. Video‐level metrics (views, likes, comment count) and comment‐level metadata (text, timestamp, reply count) were recorded.

### 2.3 Video Inclusion and Exclusion

**FIGURE 1:**
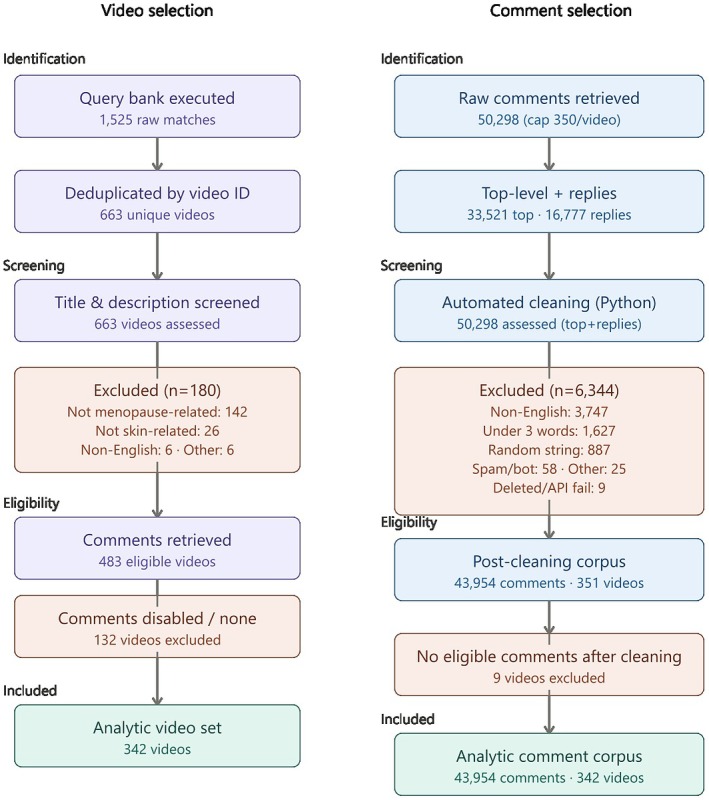
Two‐panel PRISMA flow diagram for video selection (left) and comment selection (right). All data were extracted on 14 March 2026 via the YouTube Data API v3. The comment sampling cap of 350 comments per video was applied prior to cleaning. Exclusion reasons are mutually exclusive and applied sequentially.

Of 663 deduplicated videos screened, 483 met eligibility criteria; 132 were subsequently excluded due to disabled or absent comments, leaving 351 videos for comment retrieval (Figure 1).

### 2.4 Comment Sampling and Data Cleaning

A pre‐specified cap of 350 top‐level comments per video was applied prior to cleaning; without this cap, the top 5% of videos by comment count (*n* = 18) would have accounted for 40.3% of all retrievable comments. Automated cleaning (Python) excluded non‐English comments (langdetect, confidence threshold ≥ 0.90), fragmentary comments (< 3 words), random strings, spam, and deleted content [8]. Nine videos with no eligible comments post‐cleaning were excluded, yielding a final analytic corpus of 43 954 comments from 342 videos (Figure 1). No date restriction was applied to video upload date or comment timestamp; November 2013 represents the earliest comment date identified in the dataset.

### 2.5 Video Categorization

Each video was assigned to one of four content‐type categories: (1) clinician or healthcare professional; (2) personal or lifestyle creator, encompassing patient experience and beauty or lifestyle content; (3) brand or commercial; (4) news or media. Categorization was performed automatically using a rule‐based keyword classifier applied to video title, channel name, and description text, for example, presence of “Dr.”, “DO”, “MD”, or “NP” in the channel name or title indicated healthcare professional content, while “routine,” “haul,” or “beauty” indicated personal creator content. To assess classification reliability, all videos were independently reviewed by two dermatologists, and inter‐rater agreement was quantified using Cohen's kappa, with a pre‐specified threshold of κ ≥ 0.80 required to proceed without adjudication.

### 2.6 Management Subcorpus Construction

A management subcorpus was constructed by applying pre‐specified keyword matching across 13 treatment categories (Table S1) to the full analytic corpus. The alternative/non‐evidence‐based category comprised acupuncture, gua sha, face yoga, jade roller, natural and herbal remedies, aromatherapy, homeopathic treatments, and traditional medicine practices including ayurvedic and Chinese medicine. Comments containing at least one keyword were retained; multi‐label assignment was permitted where comments referenced more than one category. This yielded 10 861 comments (24.7% of the full corpus) with 12 006 category‐level matches.

### 2.7 Sentiment Analysis

VADER (Valence Aware Dictionary and sEntiment Reasoner) sentiment analysis was applied to all comments in the full corpus and the management subcorpus [13]. VADER was selected for its validated performance on short social media text and YouTube corpora. Three analyses were conducted: temporal trend (monthly compound scores, November 2013–March 2026), sentiment by management category, and sentiment by video content type. Positive sentiment was defined as comments expressing endorsement, perceived benefit, satisfaction, or favorable views toward a treatment; negative sentiment as concern, fear, dissatisfaction, adverse experience, or skepticism; and neutral sentiment as comments with no discernible valence in either direction. Each comment was automatically assigned a compound score between −1 and + 1 by the VADER algorithm, based on the combined valence of individual words in a pre‐built lexicon adjusted for negation, intensifiers, and punctuation; no manual scoring was applied. Scores were categorized using standard VADER thresholds: positive (≥ 0.05), negative (≤ −0.05), and neutral (−0.05 to 0.05) [13]. Temporal trend reflects mean monthly compound scores, not comment volume. For HRT, an exploratory pre/post comparison used the FDA's November 2025 boxed warning removal as the index event.

### 2.8 Topic Modeling

BERTopic topic modeling [14] was applied in two stages: first to the full corpus to generate a broad thematic map, and second to the management subcorpus for treatment‐specific discourse. Full corpus topics were subsequently grouped into three superordinate thematic domains: management‐related discourse, personal narrative, and healthcare access based on semantic similarity of cluster content. Topic coherence was assessed using the C_v metric. Full BERTopic results are reported in a companion publication; findings relevant to the present study are summarized in Table S2.

### 2.9 Statistical Analysis

Between‐group comparisons were conducted using the Kruskal‐Wallis test with Dunn‐Bonferroni post hoc corrections, and temporal trends were assessed using Spearman's rank correlation (ρ). Statistical significance was set at *p* < 0.05. All analyses were performed in Python 3.11.9 using the SciPy, Pingouin, and statsmodels libraries.

## 3 Results

### 3.1 Study Corpus Overview

The analytic corpus comprised 342 videos and 43 954 comments; the earliest comment in the dataset dated to November 2013. Video uploads grew markedly over the study period, with 110 videos uploaded in 2025 alone. The majority were clinician or healthcare professional videos (*n* = 256, 74.9%), followed by personal/lifestyle creators (*n* = 73, 21.3%), brand/commercial (*n* = 9, 2.6%), and news/media (*n* = 4, 1.2%); inter‐rater agreement was near‐perfect (κ = 0.934). Overall engagement was highly variable: median views 62 046 (IQR 7768–235 639), median likes 1596 (IQR 210–6027), and median comments 100 per video (IQR 14–326).

### 3.2 Engagement Metrics by Video Content Type

Engagement metrics did not differ significantly across content‐type categories (*p* = 0.119; Table 1).

**TABLE 1:** Engagement and VADER sentiment by video content‐type category.

Category	Videos (*n*)	Comments (*n*)	Median sentiment (IQR)	Positive %	Neutral %	Negative %	Median comments per Video (IQR)
Clinician/Healthcare professional	256	35 373	0.32 (0.00–0.70)	57.7	22.2	20.1	104 (14–291)
Personal/Lifestyle creator	73	7970	0.59 (0.00–0.87)	70.6	17.1	12.3	58 (22–161)
Brand/Commercial	9	566	0.49 (0.00–0.84)	67.8	16.2	16.0	88 (50–166)
News/Media^†^	4	45	n/a	n/a	n/a	n/a	3 (2–12)

*Note:* Sentiment, Kruskal‐Wallis *p* < 0.001 (three‐group comparison excluding News/Media); Dunn‐Bonferroni post hoc, clinician vs. personal/lifestyle *p* < 0.001; clinician vs. brand *p* < 0.001; personal/lifestyle vs. brand *p* = 0.068 (ns); Engagement, Kruskal‐Wallis *p* = 0.119 (ns).

Abbreviations: IQR, interquartile range; n/a, not applicable.

^†^ News/Media excluded from inferential comparisons (*n* = 4 videos; *n* = 45 comments).

### 3.3 Management Subcorpus

**FIGURE 2:**
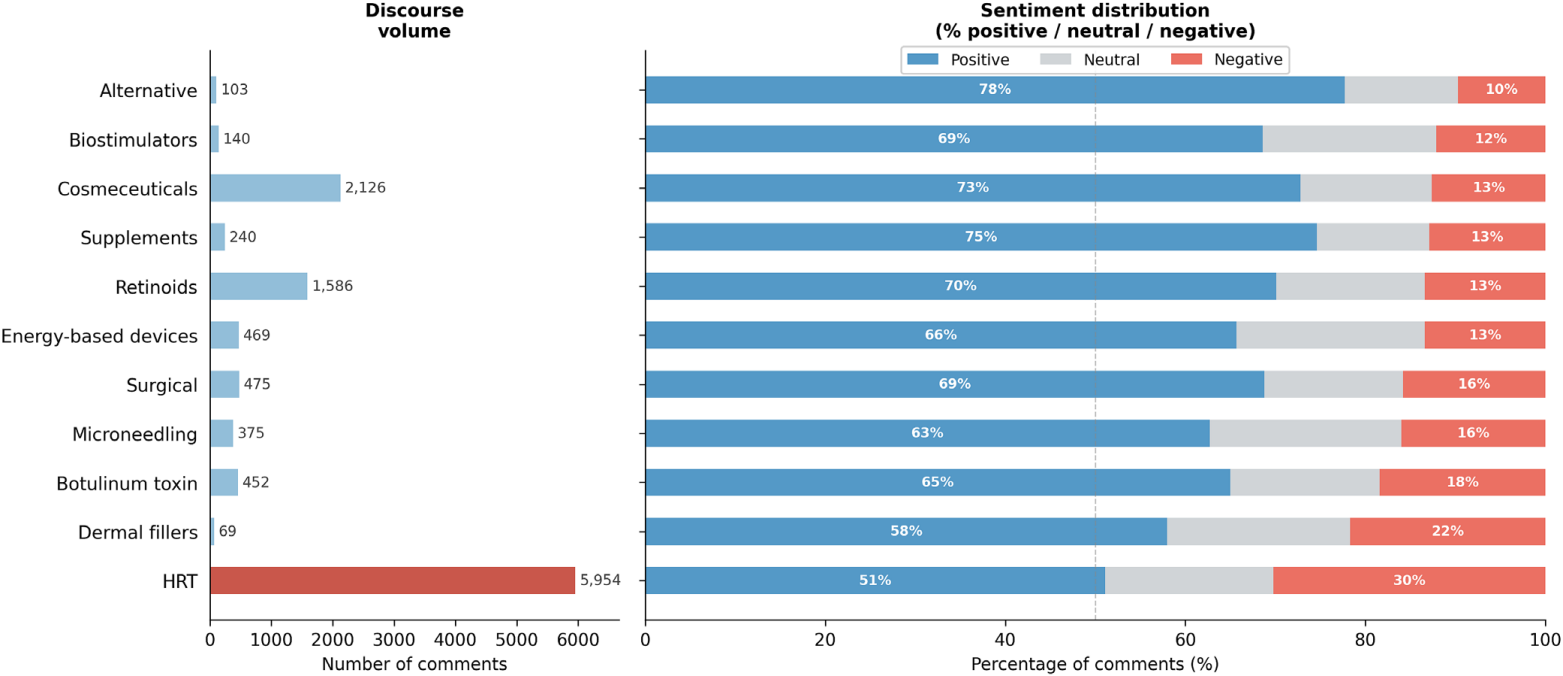
Management category discourse volume and VADER sentiment profile. Left panel: Number of comments per category; HRT (*n* = 5954) is highlighted in red to indicate its dominant discourse share and lowest sentiment. Right panel: Proportion of positive (blue), neutral (gray), and negative (red) comments per category, ordered by descending negative proportion. HRT is the only category with a negative first quartile (Q1 = −0.27); all pairwise differences between HRT and other categories were significant (*p* < 0.001) except dermal fillers (*p* = 0.157).

Of 43 954 comments, 10 861 (24.7%) contained at least one management‐related term; multi‐label assignment yielded 12 006 category‐level matches (Table 2 and Figure 2). HRT dominated the subcorpus (*n* = 5954, 54.8%), followed by cosmeceuticals (*n* = 2126, 19.6%) and retinoids (*n* = 1586, 14.6%). Procedural categories were markedly underrepresented: biostimulators (1.3%), dermal fillers (0.6%), PRP/regenerative (0.1%), and mesotherapy (0.1%).

**TABLE 2:** VADER sentiment by management category (management subcorpus, *n* = 10 861).

Category	*n*	Median VADER score (IQR)	Positive %	Neutral %	Negative %	KW Post hoc significance
HRT	5954	0.10 (−0.27 to 0.64)	51.1	18.7	30.2	vs. all (*p* < 0.001)
Cosmeceuticals	2126	0.57 (0.00 to 0.86)	72.8	14.6	12.6	—
Retinoids	1586	0.51 (0.00 to 0.85)	70.1	16.5	13.4	—
Supplements/nutraceuticals	240	0.50 (0.00 to 0.85)	74.6	12.5	12.9	—
Alternative/non‐evidence‐based	103	0.72 (0.18 to 0.89)	77.7	12.6	9.7	—
Surgical	475	0.53 (0.00 to 0.86)	68.8	15.4	15.8	—
Energy‐based devices	469	0.46 (0.00 to 0.81)	65.7	20.9	13.4	—
Botulinum toxin	452	0.43 (0.00 to 0.87)	65.0	16.6	18.4	—
Microneedling	375	0.43 (0.00 to 0.80)	62.7	21.3	16.0	—
Biostimulators	140	0.51 (0.00 to 0.85)	68.6	19.3	12.1	—
Dermal fillers	69	0.44 (0.00 to 0.92)	58.0	20.3	21.7	ns vs. HRT
PRP/Regenerative^‡^	10	0.55 (0.41 to 0.79)	80.0	20.0	0.0	—
Mesotherapy^‡^	7	0.00 (−0.01 to 0.78)	42.9	42.8	14.3	—

*Note:* H test; overall H = 726.0, *p* < 0.001. Post hoc Dunn‐Bonferroni corrections applied. HRT differed significantly from all categories (*p* < 0.001) except dermal fillers (*p* = 0.157). Among non‐HRT categories, only cosmeceuticals vs. microneedling reached significance (*p* = 0.040). — = not included in inferential comparisons.

Abbreviations: IQR, interquartile range; KW, Kruskal‐Wallis.

^‡^ PRP/Regenerative and Mesotherapy were excluded from inferential comparisons due to insufficient sample size and are presented descriptively only.

### 3.4 Sentiment Analysis

The full corpus showed predominantly positive sentiment (59.6% positive, 21.3% neutral, 19.1% negative; median compound score 0.36, IQR 0.00–0.73).

Sentiment differed significantly by video content type (*p* < 0.001; Table 1). Personal/lifestyle creators attracted the highest positivity (median 0.59, 70.6% positive), followed by brand/commercial (median 0.49, 67.8%) and clinicians (median 0.32, 57.7%). Both personal/lifestyle creators and brand/commercial channels generated significantly more positive sentiment than clinician content (*p* < 0.001 for each), while personal/lifestyle and brand/commercial channels did not differ significantly from each other (*p* = 0.068). News/media was excluded from inferential comparisons (*n* = 4 videos, *n* = 45 comments).

Within the management subcorpus, sentiment varied significantly across treatment categories (*p* < 0.001; Table 2). HRT was the sole category with a negative first quartile (Q1 = −0.27, median 0.10, 30.2% negative, 51.1% positive) and differed significantly from all other categories (all *p* < 0.001), with the exception of dermal fillers (*p* = 0.157). At the positive extreme, alternative/non‐evidence‐based approaches (median 0.72, 77.7% positive) and supplements (median 0.50, 74.6% positive) predominated. Procedural categories occupied an intermediate position (medians 0.43–0.53). Among non‐HRT categories, the only significant pairwise difference was cosmeceuticals versus microneedling, with cosmeceuticals attracting higher positivity (median 0.57 vs. 0.43; *p* = 0.040).

### 3.5 Temporal Trend

**FIGURE 3:**
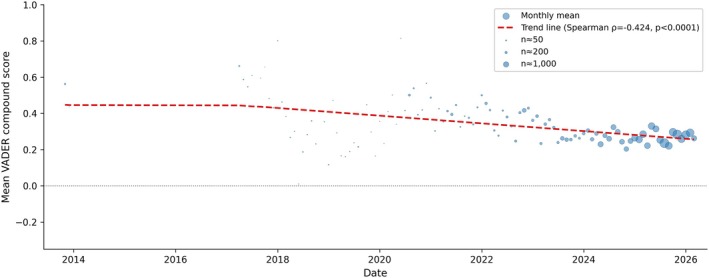
Temporal trend in mean monthly VADER compound sentiment score, November 2013 to March 2026 (*n* = 116 months). Each data point represents the mean compound score for all comments published in that calendar month. Point size is proportional to comment volume per month. The dashed line shows the Spearman regression trend (ρ = −0.424, *p* < 0.0001). Shading denotes the 95% confidence interval of a LOESS smoothing curve. Early months (2013–2019) are characterized by low comment volumes and high inter‐month volatility; the high‐volume contemporary period (2024–2026) stabilizes around a mean of 0.27.

A significant negative temporal trend in sentiment was observed across 116 months (ρ = −0.424, *p* < 0.0001; Figure 3). Monthly means ranged from −0.19 to +0.88, with high early volatility attributable to low comment volumes (< 50 comments per month before 2020). The contemporary high‐volume period (2024–2026) stabilized around a mean of 0.27.

An exploratory pre/post comparison around the FDA's November 2025 HRT boxed warning removal showed a numerically higher proportion of positive HRT comments in the post‐announcement period (53.1% positive, 29.2% negative, *n* = 1379) compared with the pre‐announcement period (50.5% positive, 30.5% negative, *n* = 4575), but this difference did not reach statistical significance (*p* = 0.126) over the 4.5‐month post‐announcement window available in this dataset.

### 3.6 Broader Discourse Themes

BERTopic modeling of the full corpus identified three dominant thematic clusters beyond treatment‐specific discourse. Management‐related commentary accounted for 26.4% of all comments (*n* = 11 595, BERTopic‐derived cluster membership and differs from the keyword‐based management subcorpus of *n* = 10 861), personal narrative and emotional sharing for 15.7% (*n* = 6914), and healthcare access concerns for 12.6% (*n* = 5532). Within the healthcare access cluster, 3.2% of all comments (*n* = 1392) reflected active information‐seeking and peer support, 3.1% (*n* = 1380) cited cost and financial barriers to treatment, and 0.4% (*n* = 159) described explicit doctor–patient communication failures including symptom dismissal or delayed diagnosis.

### 3.7 Topic Modeling and Thematic Analysis

BERTopic identified two discourse domains outside the a priori keyword framework: photoprotection (*n* = 1115, 2.5% of full corpus) and a pigmentation cluster including melasma explicitly attributed to estrogen or HRT (*n* = 280; Table S2).

## 4 Discussion

To our knowledge, this is the first study to apply comment‐centred infodemiology methodology to menopause, and specifically to menopausal skin changes and their management. The sustained growth of the corpus, with 110 videos uploaded in 2025 alone, reflects the broader mainstreaming of menopause discourse online, a shift accelerated by celebrity advocacy, policy initiatives including the UK Menopause Taskforce, and the FDA's November 2025 removal of HRT boxed warnings [12, 15]. Clinicians produced 74.9% of included videos, yet clinical credibility did not translate into more favorable viewer sentiment.

The most clinically important finding concerns HRT. Despite representing 54.8% of all management subcorpus comments, HRT carried the most negative sentiment of any category, with 30.2% of comments negative. Qualitative research has consistently documented cancer concerns, risk misperception, and limited knowledge as drivers of HRT avoidance [16, 17]; the present findings extend this by demonstrating, at scale, that these attitudes are actively expressed in online public discourse. This is particularly consequential given meta‐analytic evidence supporting HRT's beneficial effects on menopausal skin [18] and a substantially revised risk–benefit profile in the 2022 NAMS statement and 2024 WHI follow‐up [3, 19]. The FDA's November 2025 removal of HRT boxed warnings offers a natural opportunity to monitor whether regulatory action can shift entrenched public attitudes [12]; our exploratory pre/post announcement comparison showed a numerically higher but non‐significant increase in positive comments following the announcement (53.1% vs. 50.5%; *p* = 0.126), with the 4.5‐month window precluding definitive conclusions.

A parallel pattern is evident at the procedural end of the treatment spectrum. HRT, retinoids, and cosmeceuticals collectively accounted for 89.0% of management comments, while biostimulators, dermal fillers, and energy‐based devices represented fewer than 5% despite randomized trial evidence and expert consensus identifying PLLA, hyaluronic acid, and CO2 laser resurfacing among the most effective options for hormone‐related skin changes [20, 21]. This online invisibility of procedural options contrasts with real‐world patient interest: a recent international survey of 4303 perimenopausal and postmenopausal women across nine countries found that 80% and 75% were likely or very likely to seek more information on PLLA‐SCA and hyaluronic acid fillers respectively for menopause‐related skin changes, and 72% and 67% would consider requesting these treatments from a healthcare professional [22]. Such a discrepancy may partly reflect the scarcity of procedural content on YouTube rather than a lack of patient interest, reinforcing the case for greater clinician engagement in this space. At the same time, supplements and alternative/non‐evidence‐based approaches attracted the highest positivity of any category (74.6% and 77.7% respectively), despite an evidence base largely comprising small, short‐duration studies with heterogeneous endpoints and no skin‐specific regulatory approval [5, 23]. The result is an informational environment in which procedural options with established trial evidence receive little attention relative to their clinical relevance.

Clinicians produced the majority of included videos yet generated lower viewer positivity than personal/lifestyle creators, a finding that does not necessarily reflect dissatisfaction with clinical expertise. Comparable social media research suggests clinician content attracts deliberative, safety‐focused commentary while lifestyle content generates experiential responses, a structural difference in audience engagement rather than a judgment on clinical quality [10]. More concerning is the absence of a detectable sentiment difference between brand/commercial and personal/lifestyle content, consistent with documented difficulty among viewers in distinguishing commercial from independent sources [24]. These findings suggest that narrative framing and communication style shape audience engagement independently of clinical authority.

Beyond treatment‐specific discourse, 3.2% of comments reflected peer information‐seeking and 3.1% cited cost and access barriers, suggesting YouTube functions partly as a substitute for clinical contact, consistent with qualitative evidence on barriers to menopausal care [17]. A particularly actionable finding emerged from BERTopic analysis: 280 comments explicitly linked melasma or pigmentation concerns to estrogen or HRT, a cluster not previously documented in this context. Current evidence suggests a hormonal contribution to melasma risk in susceptible individuals [4, 25]. The volume of commentary suggests this concern is underaddressed and warrants proactive discussion with patients considering or using hormone therapy.

The negative temporal trend (ρ = −0.424) most plausibly reflects audience broadening rather than a genuine shift in public attitudes: before 2020, comment volumes were low and monthly sentiment highly variable, while the post‐2020 period stabilized as menopausal skin content reached a wider and more heterogeneous viewership. The comment positivity of 59.6% should additionally be read against the positivity bias of online commentary, whereby users disproportionately engage with and post positive content, the HRT negativity identified here therefore likely underestimates the true attitudinal gap [26, 27].

## 5 Limitations

This study is limited to English‐language YouTube content, and commenter demographics cannot be verified from metadata. VADER may misclassify hedged clinical language, and its agreement with manual coding is only moderate in health‐related settings; keyword‐based subcorpus construction may additionally miss colloquial treatment references. The 4.5‐month post‐FDA announcement window limits conclusions about regulatory impact on public sentiment; extended follow‐up is warranted as longitudinal data accumulate.

## 6 Conclusion

Public discourse on menopausal skin management on YouTube reveals a clinically consequential inversion: HRT generates the most negative commentary of any treatment category, while supplements and alternative approaches attract positivity disproportionate to their evidence base, and procedural interventions with growing trial‐level support remain largely absent from viewer discourse. For dermatologists engaging in digital health communication, addressing HRT safety misconceptions and increasing the visibility of evidence‐based procedural options require not only clinical accuracy but communication strategies that match the accessibility of the content currently shaping patient beliefs.

## Author Contributions

Esin Diremsizoglu, Emre Akif Arslan, Abdullah Demirbas, and Sabrina G. Fabi contributed to the study conception and design. Esin Diremsizoglu, Emre Akif Arslan collected and analyzed the data. Esin Diremsizoglu, Emre Akif Arslan, Abdullah Demirbas, and Sabrina G. Fabi interpreted the findings, and contributed to drafting and revising the manuscript. All authors have read and approved the final manuscript.

## Funding

The authors have nothing to report.

## Ethics Statement

This study analyzed publicly available YouTube viewer comments and required no ethics approval. No personally identifiable information was recorded; usernames were not collected, and any identifiable details encountered during data extraction were pseudonymised prior to analysis.

## Conflicts of Interest

The authors declare no conflicts of interest.

## Supporting information


**Table S1:** Management‐specific subcorpus keyword framework.
**Table S2:** BERTopic‐identified discourse domains not captured by the a priori keyword framework.

## Data Availability

The data that support the findings of this study are available from the corresponding author upon reasonable request.
