# Supplementary material for: Public Discourse on Menopausal Skin Management: A YouTube Infodemiology Study of Treatment Perceptions, Sentiment, and Unmet Needs Across 43 954 Viewer Comments
Source: J Cosmet Dermatol. 2026 Jul 27;25(8):e71101. doi: 10.1111/jocd.71101 (PMC13403088; doi:10.1111/jocd.71101)
Supplement: Supplementary file 1 — Table S1: Management‐specific subcorpus keyword framework. Table S2: BERTopic‐identified discourse domains not captured by the a priori keyword framework. [file JOCD-25-e71101-s001.docx]

**Supplementary Table 1. Management-specific subcorpus keyword framework.**

| **Category** | **Keywords** |
| --- | --- |
| Systemic HRT | hrt, mht, hormone replacement, hormone therapy, menopausal hormone, estradiol, oestradiol, estrogen, oestrogen, progesterone, progestogen, progestin, transdermal patch, bioidentical, premarin, vivelle, climara, divigel, estrogel, prometrium, utrogestan, tibolone, duavee, bijuva, evamist, combipatch, activella, angeliq, raloxifene, serm, selective estrogen receptor modulator |
| Topical estrogen & phytoestrogens* | topical estrogen, vaginal estrogen, estriol, mep, methyl estradiolpropanoate, compounded estrogen, topical estradiol, phytoestrogen cream, isoflavone, genistein, soy isoflavone, topical phytoestrogen, resveratrol cream |
| Topical retinoids | retinol, retinoid, tretinoin, retinoic acid, retinaldehyde, adapalene, tazarotene, differin, retin-a, renova, granactive retinoid |
| Cosmeceuticals / skincare actives | hyaluronic acid, vitamin c serum, ascorbic acid, niacinamide, skin peptide, ceramide cream, spf cream, sunscreen, azelaic acid, glycolic acid, lactic acid, squalane oil, growth factor serum, egf serum, topical exosome, exosome serum, resveratrol serum, tranexamic acid, retinol |
| Energy-based devices | laser treatment, laser resurfacing, ipl treatment, intense pulsed light, radiofrequency treatment, radiofrequency skin, hifu treatment, mfu-v, microfocused ultrasound, ultherapy, thermage, morpheus8, fractional laser, co2 laser, erbium laser, picosecond laser, fraxel, clear and brilliant, halo laser, moxi laser, led therapy, red light therapy, sofwave, emface, forma rf, inmode treatment, bbl treatment, photofacial, profound rf |
| Microneedling | microneedling, micro needling, collagen induction therapy, dermaroller, dermapen, skinpen, aquagold |
| Dermal fillers | dermal filler, skin filler, hyaluronic filler, juvederm, restylane, belotero, teosyal, lip filler, cheek filler, tear trough filler |
| Biostimulators | biostimulator, sculptra, plla, poly-l-lactic acid, radiesse, calcium hydroxylapatite, caha, hyperdiluted radiesse, profhilo, polynucleotide injection, pdrn injection, collagen stimulator, jalupro, rejuran, nucleofill, lanluma, ellanse |
| Botulinum toxin | botox, botulinum toxin, dysport, xeomin, jeuveau, daxxify |
| PRP / Regenerative | prp treatment, platelet rich plasma, prp facial, exosome therapy |
| Surgical | facelift, face lift, blepharoplasty, eyelid surgery, brow lift, thread lift, pdo thread, neck lift, fat transfer, fat grafting, cosmetic surgery, plastic surgery |
| Supplements / nutraceuticals | collagen supplement, collagen powder, collagen peptide, marine collagen, bovine collagen, omega 3 supplement, fish oil supplement, vitamin d supplement, vitamin e supplement, zinc supplement, biotin supplement, black cohosh, evening primrose oil, beauty supplement, skin supplement, astaxanthin, pycnogenol, glutathione supplement, coenzyme q10, resveratrol supplement |
| Non-evidence-based / alternative | natural remedy, herbal remedy, homeopathic treatment, ayurvedic, chinese medicine, acupuncture, aromatherapy, jade roller, gua sha, face yoga, lymphatic drainage facial |
| Mesotherapy | mesotherapy, meso injection, skin booster injection, skinbooster |

**Topical estrogen & phytoestrogens category yielded no keyword matches in the analytic corpus and is therefore excluded from sentiment analysis (Table 2).*

**Supplementary Table 2. BERTopic-identified discourse domains not captured by the a priori keyword framework.**

| **Domain** | **Discourse Cluster** | **n** | **% of Full Corpus (N = 43,954)** |
| --- | --- | --- | --- |
| Photoprotection | Sun protection and photoprotection | 1,115 | 2.5 |
| Pigmentation | Hyperpigmentation and dark spots (general) | 508 | 1.2 |
| Pigmentation | Melasma attributed to oestrogen or HRT | 280 | 0.6 |
| Pigmentation | Hormonal acne and perimenopausal breakouts | 312 | 0.7 |
| Healthcare access | Information-seeking and peer support | 1,392 | 3.2 |
| Healthcare access | Cost and financial access barriers | 1,380 | 3.1 |
| Healthcare access | Doctor–patient communication gap | 159 | 0.4 |

*BERTopic = Bidirectional Encoder Representations from Transformers topic modelling. n values reflect keyword-confirmed counts within the corpus following BERTopic cluster identification; overlap between categories is possible. Percentage calculated against full corpus (N=43,954).*
